# Long-term physical training in adolescent sprint and middle distance swimmers alters the composition of circulating T and NK cells which correlates with soluble ICAM-1 serum concentrations

**DOI:** 10.1007/s00421-021-04660-1

**Published:** 2021-03-11

**Authors:** Hannah L. Notbohm, Moritz Schumann, Stefan Fuhrmann, Jan Klocke, Sebastian Theurich, Wilhelm Bloch

**Affiliations:** 1 Department of Molecular and Cellular Sports Medicine, German Sport University Cologne, Am Sportpark Müngersdorf 6, 50933 Cologne, Germany; 2 Olympic Training Facility Hamburg/Schleswig-Holstein, Hamburg, Germany; 3 Olympic Training Facility NRW/Rhein-Ruhr, Essen, Germany; 4 Cancer and Immunometabolism Research Group, Department of Medicine III, LMU University Hospital Munich, Munich, Germany; 5 Gene Center, Ludwig-Maximilians-University Munich, Munich, Germany

**Keywords:** Cell adhesion molecules, Cell trafficking, CD45RA, Lymphocytes, Immune system, Training intensity distribution

## Abstract

**Purpose:**

It remains unknown how different training intensities and volumes chronically impact circulating lymphocytes and cellular adhesion molecules. First, we aimed to monitor changes in NK and T cells over a training season and relate these to training load. Second, we analyzed effects of training differences between swimmers on these cells. Finally, we examined if changes in lymphocytes were associated with sICAM-1 concentrations.

**Methods:**

We analyzed weekly training volume, training intensity, proportions of T and NK cells and serum sICAM-1 in eight sprint (SS) and seven middle-distance swimmers (MID) at three points over a 16-week training period: at the start (*t*_0_), after 7 weeks of increased training load (*t*_7_) and after 16 weeks, including 5-day taper (*t*_16_).

**Results:**

Training volume of all swimmers was statistically higher and training intensity lower from *t*_0_–*t*_7_ compared to *t*_7_–*t*_16_ (*p* = 0.001). Secondly, training intensity was statistically higher in SS from *t*_0_–*t*_7_ (*p* = 0.004) and *t*_7_–*t*_16_ (*p* = 0.015), while MID had a statistically higher training volume from *t*_7_–*t*_16_ (*p* = 0.04). From *t*_0_–*t*_7_, NK (*p* = 0.06) and CD45RA^+^CD45RO^+^CD4^+^ cells (*p* < 0.001) statistically decreased, while CD45RA^−^CD45RO^+^CD4^+^ cells (*p* = 0.024) statistically increased. In a subgroup analysis, SS showed statistically larger increases in NK cells from *t*_7_–*t*_16_ than MID (*p* = 0.012). Lastly, sICAM-1 concentrations were associated with changes in CD45RA^−^CDRO^+^CD4^+^ cells (*r* = − 0.656, *p* = 0.08).

**Conclusion:**

These results indicate that intensified training in swimmers resulted in transient changes in T and NK cells. Further, NK cells are sensitive to high training volumes. Lastly, sICAM-1 concentrations may be associated with the migration and maturation of CD4^+^ cells in athletes.

## Introduction

Bouts of intensive exercise and strenuous periods of training have previously been shown to lead to changes in the populations of circulating lymphocytes in the bloodstream, while periods with reduced training load may reverse some of these changes (Cosgrove et al. 2012; Gleeson et al. 1995; Mujika et al. 1996; Rama et al. 2013; Teixeira et al. 2014). For example, previous training studies have consistently shown a decrease in numbers of natural killer (NK) cells as a result of long periods of training (> 12 weeks) (Gleeson et al. 1995, 2000; Rama et al. 2013). In contrast, findings concerning T cells are more heterogeneous. In a study examining young adult swimmers over a 29-week training period, populations of naïve CD4^+^ (CD45RA^+^ CD45RO^−^ CD4^+^), transitional CD4^+^ (CD45RA^+^ CD45RO^+^ CD4^+^) and memory CD4^+^ (CD45RA^−^ CD45RO^+^ CD4^+^) cells changed in relation to in water training (Teixeira et al. 2014), while in a 6-month training period of adult triathletes leading up to an Ironman, changes were mainly found in proportions of transitional CD4^+^ (CD45RA^+^ CD45RO^+^ CD4^+^) cells (Cosgrove et al. 2012). Moreover, regulatory T cells (Treg) have been shown to be sensitive to periods of increased high-intensity training. For example, numbers of Treg cells were shown to statistically increase after 1 week of high-intensity training in adult hockey players (Weinhold et al. 2016). Thus, the most responsive cells to exercise tend to have stronger cytotoxic functions (Witard et al. 2012), express phenotypes that are strongly associated with tissue migration (Simpson et al. 2006), tend to be highly responsive to catecholamines due to a high expression of β-adrenoreceptors (Krüger et al. 2007) and express more mature phenotypes (Simpson et al. 2007). Therefore, the distribution and function of different subclasses of lymphocytes are essentially altered in response to exercise.

However, the mechanisms underlying the increased levels of lymphocyte trafficking induced by exercise have not been fully understood. The mobilization of lymphocytes into the bloodstream from peripheral lymphoid organs or marginated pools in the endothelium may be mediated by increased levels of catecholamines (Krüger et al. 2007) or induced shear stress from the larger cardiac output and increased blood flow during exercise (Shephard 2003). It is possible that exercise-induced egress from the blood may reflect mobilization of lymphocytes into peripheral tissues and organs to increase immune surveillance (Krüger et al. 2007; Krüger and Mooren 2007). This is a critical process for the efficiency of the immune system to promote cell-to-cell interactions and, if necessary, generate adequate immune responses. These processes of migration and redistribution are regulated by the secretion of hormones, especially catecholamines and glucocorticoids (Dhabhar 2014; Krüger et al. 2007), secretion of cytokines and differential expression of chemokines and cell adhesion molecules in different target tissues (Constantin et al. 2000). Therefore, exercise-induced lymphocyte mobilization is particularly dependent on the expression of cell surface adhesion molecules and their counterreceptors (Shephard 2003).

Migration of lymphocytes across the endothelium is a multistep process and molecules like intercellular adhesion molecule 1 (ICAM-1) play a major role in the firm adhesion of lymphocytes to the endothelium and pave the way for migration (Hubbard and Rothlein 2000). Catecholamines have previously been shown to play a role in the regulation of ICAM-1 in the cell adhesion of lymphocytes to the endothelium, with increased levels of catecholamines inducing a shedding of ICAM-1 and an increase in soluble ICAM-1 (sICAM-1) in the blood (Rehman et al. 1997). This in turn leads to a detachment of cells from the endothelium and a competitive inhibition of binding to adhesion receptors of neighboring cells. Therefore, increased levels of sICAM-1 may inhibit the extravasation of lymphocytes into peripheral tissues. After an acute bout of exercise, levels of sICAM-1 temporarily increase (Akimoto et al. 2002; Nielsen and Lyberg 2004; Rehman et al. 1997) but little is known about the effect of longer training and recovery periods on levels of sICAM-1 and the relationship to changes in lymphocyte populations.

As proper immune responses are dependent on the ability of lymphocytes to migrate into surrounding tissues and sites of inflammation (Pedersen and Hoffman-Goetz 2000), it is important to determine the response of lymphocytes to changes in training intensity and volume and investigate the underlying mechanisms. To date, it remains unclear how periods of training at different intensities and volumes chronically impact populations of circulating T and NK cells and whether these changes are influenced by cellular adhesion molecules. Therefore, the aims of this prospective controlled study were threefold. First, we aimed to monitor changes in NK and T cells over the course of a 16-week training season and relate these to training load. Second, we analyzed the effects of differences in training volume and intensity between sprint (SS) and middle (MID) distance swimmers on populations of NK and T cells. Finally, it was examined if changes in lymphocyte populations were associated with changes in sICAM-1.

## Methods

### Study design and training intervention

Training volume and intensity were monitored for a group of SS and MID swimmers during 16 weeks of the swimming season from August to December. Blood was collected at three time points throughout the season: before the start of the season after 3 weeks training of freely chosen volume and intensity (*t*_0_), after the first 7 weeks of increased training load (*t*_7_) and in the last week after 16 weeks of training and a pre-competition tapering period (*t*_16_). In-water training consisted of 7–8 weekly sessions of 1.5 h for SS and 2 h for middle distance swimmers, including both low intensity continuous as well as high-intensity interval training. In addition, dry land strength training was performed two or three times a week for MID and SS swimmers, respectively. Training volume was recorded as kilometers swum per week. To assess training intensity, a three-training zone model was established (Seiler and Kjerland 2006). Training zones were determined in relation to maximal swimming speed (*V*_max_): below 75% *V*_max_, between 75 and 85% *V*_max_ and above 85% *V*_max_. Subjective training load was assessed using the session RPE method first developed by Foster et al. (1995). Athletes rated the intensity of the entire training session on the 6–20 BORG scale and this intensity value was then multiplied by session duration (minutes), creating a single measure of internal training load (arbitrary units, AU).

### Participants

A team of 15 well-trained adolescent swimmers volunteered to participate in this study. The swimmers were part of a group training at the Olympic Training Centre and regularly competed at the national level, with a training history of at least 5 years. The swimmers were classified as SS (50/100 m distance; *n* = 8, age: 15.3 ± 1.2 years, height: 177.2 ± 10.5 cm and body mass: 68.2 ± 11.5 kg) or MID (200/400 m distance; *n* = 7, 14.4 ± 1 years, height: 177.3 ± 4 cm and body mass: 66.1 ± 7.2 kg). All participants were informed about possible risks of the study and written informed consent was provided both by the participants as well as their legal guardian prior to the start of the study. The study was conducted according to the declaration of Helsinki and ethical approval was granted by the institutional review board (078/2018).

### Blood sampling

Blood samples were collected in the morning after an overnight fast and at least 24 h of rest. Blood was collected from the antecubital vein into sterile vacutainers, either K_2_-EDTA tubes for blood cells counts or serum separation tubes (BD Vacutainer, Beckton, Dickinson, Heidelberg, Germany). Blood was transported on ice and serum was centrifuged for 10 min at 2195 ×*g* 12 h after collection and stored at -80 °C for further analysis. EDTA blood was kept on a roller until FACS analysis was performed 24 h after blood collection.

### Analytical procedures

We analyzed T and NK cell subpopulations from whole blood by multi-color flow cytometry (FACSArray, Becton Dickinson, Heidelberg, Germany). Gating strategies were applied as previously described elsewhere (Wenning et al. 2013). The following antibodies were used: CD45 APC-Cy7 (clone 2D1), CD4 APC (clone SK3), CD3 PE-Cy7 (clone SK7), CD19 APC (clone SJ25C1), CD8 PE (clone SK1), CD45RA PE (clone HI100), CD45RO PE-Cy7 (clone UCHL1), CD127 PE (clone HIL7RM2) and CD25 APC (clone M-A251) (all antibodies: BD, Heidelberg, Germany). In addition, serum concentrations of sICAM-1 were determined by enzyme-linked immunosorbent assays (ELISA) in a laboratory specialized in clinical routine diagnostics (Labor Dr. Wisplinghoff, Cologne, Germany).

### Statistical analysis

Data were firstly analyzed for the whole group of swimmers (pooled analysis) and in a second step for the subgroups of SS and MID separately. Results are presented as mean ± SD. Due to the limited sample sizes, data were analyzed by using non-parametric tests. Differences between time points were assessed using the Wilcoxon Signed Rank Test and a Bonferroni correction was applied for *p *values, by multiplying all pair-wise p-values with the number of comparisons conducted for each variable (i.e. *t*_0_–*t*_7_, *t*_0_–*t*_16_ and *t*_7_–*t*_16_, respectively). Between-group comparisons of SS and MID were analyzed by the Mann–Whitney *U* test. Data from *t*_7_ and *t*_16_ were normalized to baseline values. The effect size *r* (ES) was calculated from the *z*-score and number of observations *n*, with ES > 0.1 being a small effect, ES > 0.3 being a medium effect and ES > 0.5 being a large effect (Rosenthal and DiMatteo 2001). To evaluate associations between dependent variables, Spearman Correlation Coefficients were calculated. Statistical significance for all tests was set at *p* ≤ 0.05. All data were analyzed using IBM SPSS Predictive Analytics (version 26.0, IBM Inc., Chicago, USA).

## Results

### Training

Mean weekly training volume, the proportion of training above 85% *V*_max_ and mean weekly session RPE are shown in Table 1. Differences in these variables were observed between time periods for pooled analysis (all *p* < 0.001), SS (*p* = 0.016, *p* = 0.008, *p* = 0.008, respectively) and MID (all *p* = 0.016). Statistical differences were also found for between groups from *t*_0_ to *t*_7_ for the proportion of training above 85% *V*_max_ (*p* = 0.001) and weekly session RPE (*p* = 0.001) and from *t*_7_ to *t*_16_ for mean weekly training volume (*p* = 0.04), training above 85% *V*_max_ (*p* = 0.015) and weekly session RPE (*p* = 0.001).

**Table 1:** Analysis of training volume, training intensity and subjective training load

	Pooled analysis		Sprint		Middle distance	
	*t*_0_–*t*_7_	*t*_7_–*t*_16_	*t*_0_–*t*_7_	*t*_7_–*t*_16_	*t*_0_–*t*_7_	*t*_7_–*t*_16_
km/week	34.0 ± 3.7	28.7 ± 3.0*	32.5 ± 3.9*	26.6 ± 2.6*^#^	35.7 ± 2.8*	31.1 ± 1.3*^#^
% training above 85% *V*_max_	5.6 ± 1.3	8.3 ± 0.7*	6.8 ± 0.8*^#^	8.5 ± 0.4*^#^	4.5 ± 0.5*^#^	7.8 ± 0.5*^#^
Weekly session RPE	1458 ± 167	1380 ± 181*	1321 ± 48*^#^	1231 ± 62*^#^	1716 ± 53*^#^	1653 ± 47*^#^

Data are mean ± SD

*Statistical difference between time periods

^#^Statistical difference between groups

### Pooled analysis of changes in T and NK cells

**Fig. 1:**
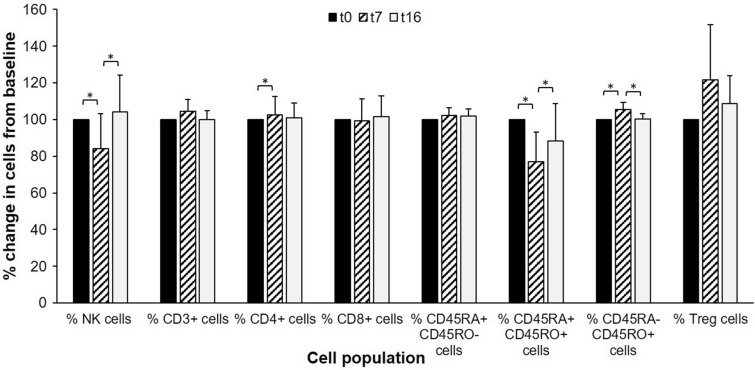
Pooled changes in proportions of NK cells of lymphocytes, CD3 + cells of lymphocytes, CD4 + cells of CD3 + cells, CD8 + cells of CD3 + cells, naive (CD45RA + CD45RO-) cells of CD4 + cells, transitional (CD45RA + CD45RO +) cells of CD4 + cells, memory (CD45RA- CD45RO +) cells of CD4 + cells and Treg cells of CD3 + cells. * Statistical difference between time points

Changes in NK cells and T cells subsets throughout the training season are displayed in Table 2 and Fig. 1. Proportions of NK cells statistically decreased from *t*_0_ to *t*_7_ (*p* = 0.006, ES = 0.52) and statistically increased from *t*_7_ to *t*_16_ (*p* = 0.006, ES = 0.53). Proportions of CD4^+^ cells statistically increased from *t*_0_ to *t*_7_ (*p* = 0.036, ES = 0.45) and remained statistically unchanged thereafter (*p* = 0.366, ES = 0.29). Proportions of transitional CD4^+^ (CD45RA^+^ CD45RO^+^ CD4^+^) cells statistically decreased from *t*_0_ to *t*_7_ (*p* < 0.001, ES = 0.61) and statistically increased from *t*_7_ to *t*_16_ (*p* = 0.003, ES = 0.57). Proportions of memory CD4^+^ (CD45RA^−^ CD45RO^+^ CD4^+^) cells statistically increased from *t*_0_ to *t*_7_ (*p* = 0.024, ES = 0.47) and statistically decreased from *t*_7_ to *t*_16_ (*p* < 0.001, ES = 0.59). The increase in proportions of Treg cells from *t*_0_ to *t*_7_ nearly reached statistical significance (*p* = 0.06, ES = 0.42), but was not maintained thereafter (*p* = 0.492, ES = 0.26). Proportions of CD3^+^ cells, CD8^+^ cells and naïve CD4^+^ (CD45RA^+^ CD45RO^−^ CD4^+^) cells remained statistically unaltered throughout the training period.

**Table 2:** Populations of lymphocytes and serum concentration of sICAM-1 in the pooled analysis, sprint (SS) and middle distance (MID) swimmers

	Pooled analysis			SS			MID		
	*t*_0_	*t*_**7**_	*t*_16_	*t*_0_	*t*_7_	*t*_16_	*t*_0_	*t*_7_	*t*_16_
% Lymphocytes of total cells	35.6 ± 6.1	32.4 ± 6.8	35.6 ± 7.9	35.7 ± 6.4	34.4 ± 6.9	36.7 ± 5.9	39.0 ± 6.7	30.3 ± 6.5	34.4 ± 10.1
% CD3 + cells of lymphocytes	64.0 ± 5.3	66.7 ± 4.4	63.9 ± 4.4	65.5 ± 4.9	67.5 ± 4.6	63.5 ± 4.8	62.3 ± 5.5	65.7 ± 4.4	64.3 ± 4.3^†^
% CD4 + of CD3 + cells	54.1 ± 6.1	55.45 ± 8.0*	54.6 ± 7.6	53.6 ± 4.7	54.1 ± 9.1	52.7 ± 8.2	54.7 ± 7.7	57.1 ± 6.7	56.8 ± 6.7^†^
% CD8 + of CD3 + cells	35.8 ± 5.4	35.5 ± 6.9	36.2 ± 6.0	36.6 ± 6.3	37.0 ± 8.3	38.2 ± 6.7	34.9 ± 4.6	33.7 ± 4.5	33.7 ± 4.1
% NK cells of lymphocytes	15.4 ± 4.8	12.7 ± 5.0*	15.3 ± 4.4^#^	12.8 ± 4.6	10.7 ± 3.6	14.6 ± 4.9^#^	18.2 ± 6.0	15.1 ± 5.5	16.2 ± 4.0
% CD45RA- CD45RO + cells of CD4 + cells	35.2 ± 4.9	37.0 ± 5.2*	35.2 ± 5.3^#^	35.6 ± 5.6	37.2 ± 6.2	35.5 ± 5.8	34.6 ± 4.4	36.8 ± 4.4*	34.9 ± 5.0^#^
% CD45RA + CD45RO + cells of CD4 + cells	9.0 ± 2.5	5.8 ± 1.3*	7.8 ± 2.2^#^	8.3 ± 2.1	5.0 ± 1.1*	7.0 ± 2.2^#^	9.8 ± 2.7	6.8 ± 0.8	8.7 ± 2.0
% CD45RA + CD45RO- cells of CD4 + cells	55.8 ± 5.8	57.0 ± 5.7	57.0 ± 6.4	56.1 ± 6.8	57.6 ± 6.7	57.5 ± 7.0	55.6 ± 4.8	56.3 ± 4.7	56.4 ± 6.0
% Treg cells of CD3 + cells	5.1 ± 0.8	6.2 ± 1.5	5.6 ± 1.3	5.0 ± 0.7	5.9 ± 1.7	5.3 ± 1.1	5.3 ± 0.9	6.5 ± 1.4	5.9 ± 1.4
sICAM (ng ml^−1^)	200.7 ± 89.0	209.7 ± 136.8	255.8 ± 131.8	178.8 ± 47.3	245.2 ± 164.2	244.5 ± 115.5	225.8 ± 120.3	169.2 ± 92.8	268.7 ± 156.9

Data are mean ± SD. *t*_0_: after the summer break and a few weeks of freely chosen training volume, *t*_7_: after 7 weeks of intensified training, *t*_16_: after 16 weeks and a pre competition taper period

*Statistically significant change from *t*_0_ to *t*_7_

^#^Statistically significant change from *t*_7_ to *t*_16_

^†^Statistically significant change from *t*_0_ to *t*_16_

### Changes in NK cells in sprint and middle-distance swimmers

**Fig. 2:**
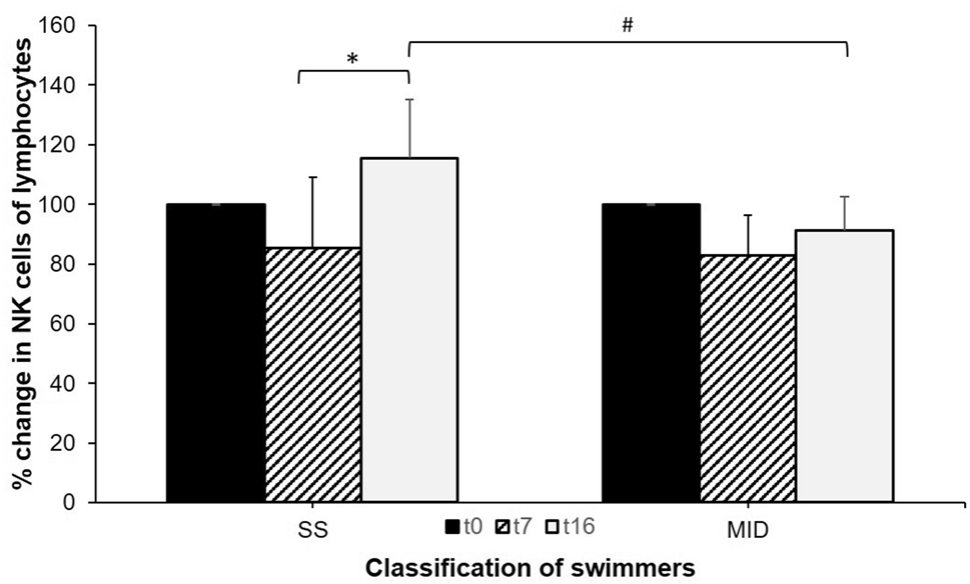
Changes in proportions of NK cells over the training season. *Statistical difference between time points, #statistical between-group difference

No statistical between-group differences were found for any cells at all timepoints apart from for NK cells. The change of proportions in NK cells from *t*_7_ to *t*_16_ was statistically larger in SS compared to MID (+ 40.0 ± 24.2% vs + 11.4 ± 15.6%, *p* = 0.012, ES = 0.69) (Fig. 2).

### Changes in sICAM-1

**Fig. 3:**
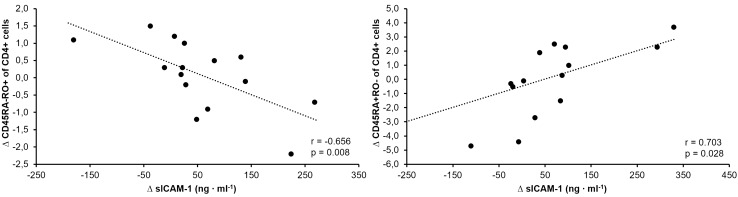
Correlation between the changes in sICAM-1 and CD45RA- CD45RO + cells over the training period (*t*_0_–*t*_16_) (**a**) and changes in sICAM-1 and CD45RA- CD45RO + cells from *t*_7_–t_16_ (**b**)

sICAM-1 remained statistically unaltered throughout the entire training period in both the pooled analysis but also the subgroup analysis. For the pooled analysis, changes in sICAM-1 were associated with changes in proportions of memory CD4^+^ (CD45RA^−^ CD45RO^+^ CD4^+^) cells from *t*_7_ to *t*_16_ (*r* = − 0.589, *p* = 0.002) and over the entire training period (*r* = − 0.656, *p* = 0.008, Fig. 3). Similarly, changes in sICAM-1 were also associated with changes in proportions of naïve CD4^+^ (CD45RA^+^ CD45RO^−^ CD4^+^) cells from *t*_7_ to *t*_16_ (*r* = 0.703, *p* = 0.028, Fig. 3). From t_7_ to t_16_, changes in sICAM-1 were associated with mean weekly training volume (*r* = 0.645, *p* = 0.009) and proportion of training above 85% *V*_max_ (*r* = 0.524, *p* = 0.045).

## Discussion

This study aimed at assessing the changes in subpopulations of T and NK cells in relation to changes in training over a 16-week period in swimmers and further assessing the influence of different training volumes and intensities (i.e. SS and MID swimmers). Furthermore, we examined whether changes in these cells are associated with changes in sICAM-1. Mean weekly training volume, training intensity and subjective training load increased through the first seven weeks of training. While training intensity was higher in the second period of training, mean weekly training volume and subjective training load decreased. SS performed a higher training intensity throughout the training period, while MID performed a higher training volume in the second period of training. Our pooled analysis revealed a statistical reduction in proportions of NK cells, and transitional CD4^+^ (CD45RA^+^ CD45RO^+^ CD4^+^) cells, while proportions of memory CD4^+^ (CD45RA^−^ CD45RO^+^ CD4^+^) cells statistically increased after the initial period of high training load. These changes statistically reversed following the second period with a decreased training load and proportions of lymphocytes returned to values similar to baseline. However, proportions of NK cells were statistically increased in SS compared to MID at the end of the training period. No statistical changes were found in serum concentration of sICAM-1 over the training period, however, changes in sICAM-1 were associated with changes in proportions of memory CD4^+^ (CD45RA^−^ CD45RO^+^ CD4^+^) cells in the second nine weeks of training and over the complete training period.

Previous studies have clearly indicated that an increased training load may induce a shift towards more differentiated cells in subclasses of CD4^+^ cells (Cosgrove et al. 2012; Teixeira et al. 2014), possibly because of increased encounters of naïve CD4^+^ (CD45RA^+^ CD45RO^−^ CD4^+^) with pathogens due to increased ventilation, eliciting their differentiation and proliferation into effector memory cells. In a 6-month training period of adult triathletes leading up to an Ironman, naïve CD4^+^ (CD45RA^+^ CD45RO^−^ CD4^+^) cells decreased and transitional CD4^+^ (CD45RA^+^ CD45RO^+^ CD4^+^) cells increased during the high-intensity training phase and also further after the competition (Cosgrove et al. 2012). Similarly, our data indicated a shift towards a more differentiated CD4^+^ cell phenotype after a period of intensified training, as the proportion of memory CD4^+^ (CD45RA^−^ CD45RO^+^ CD4^+^) cells statistically increased (+ 5.4 ± 3.8%), however, the proportion of transitional CD4^+^ (CD45RA^+^ CD45RO^+^ CD4^+^) cells statistically decreased (− 33.1 ± 16.2%). Prieto-Hinojosa et al. (2014) showed that thymic output of adult athletes is reduced. Therefore, if periods of high training load induce a shift towards a more differentiated phenotype of CD4^+^ cells, it would be expected that the proportion of naïve cells decreased in our study. However, in contrast to the aforementioned studies, we did not observe a statistical change in proportions of naïve CD4^+^ (CD45RA^+^ CD45RO^−^ CD4^+^). Whether this may be due to the adolescence of swimmers in contrast to the older populations investigated in other studies, remains to be investigated.

The decrease of proportions of NK cells after the initial seven weeks of training (− 15.8 ± 19.0%) is in line with previous studies that have shown similar reductions following periods of intensified training in young adult swimmers (Gleeson et al. 1995, 2000; Rama et al. 2013). A depletion in the proportion of these cells in the blood may be attributed to an egress into tissues requiring immune surveillance. This was previously for example shown for the lung, which has an increased probability of contacting potentially harmful agents due to the increase in ventilation (Krüger et al. 2007), and muscles, where an egress of NK cells may assist in repairing damaged tissue after vigorous exercise (Shephard and Shek 1999). However, the mechanisms and sites of migration have not been fully elucidated.

Interestingly, our data indicate that a training phase with reduced training volume and a pre-competition taper resulted in a recovery of circulating lymphocyte proportions, confirming the observation in other studies that the exercise-induced suppression of circulating lymphocytes is transient and reversible by a recovery phase (Mujika et al. 1996; Rama et al. 2013). However, differences in the recovery of proportions of NK cells were observed between SS and MID after 16 weeks of training, with SS showing a statistically larger increase in proportions of NK cells than MID. This difference is most likely due to the statistical difference in training volume between groups during the latter half of the training period, as proportions of NK cells showed a similar decrease in both groups during the initial seven weeks of training where training volume did not statistically differ between the two groups. In the second training period, training volume was statistically higher in MID compared to SS (31.3 ± 1.3 km vs. 26.6 ± 2.6 km). Therefore, it can be speculated that a higher training volume may account for the egress of NK cells from the bloodstream.

NK cell migration and infiltration of tissues and organs that require immune surveillance after exercise has been shown to be dependent on interleukin 6 (IL-6) (Pedersen et al. 2016), which is a cytokine mainly released from contracting muscle in relation to glycogen depletion (Steensberg et al. 2001). While the release of IL-6 is also dependent on the training intensity, it has been suggested that exercise duration and training volume have a greater impact on the size of the IL-6 response to exercise (Fischer 2006). As middle-distance swimmers showed a higher training volume in the second 9 weeks of training and their in water training was longer, it could be possible that consistently more IL-6 was released during training sessions, causing a larger egress of NK cells from the bloodstream, which resulted in lower proportions of circulating NK cells at rest. However, as concentrations of IL-6 were not assessed, this hypothesis remains speculative.

To further the understanding of increased lymphocyte trafficking, sICAM-1 concentrations were determined but these showed no statistical change throughout the training period. Therefore, as sICAM-1 serum concentrations and ICAM-1 expression on activated endothelium have been shown to be proportional (Leeuwenberg et al. 1992), prolonged periods of training do not seem to have an effect on the regulation of ICAM-1 and the altered proportions of circulating lymphocytes cannot be related to changes in ICAM-1 expression and altered endothelial activation. One important consideration however, is the aspect of time. Changes in concentrations of soluble factors like sICAM-1 are an immediate result of acute physical stress and act in the regulation of other processes, like the migration of lymphocytes. These effects are, however, of short duration. Previous studies have shown concentrations of sICAM-1 to return to baseline one hour after exercise (Bartzeliotou et al. 2007; Rehman et al. 1997; Simpson et al. 2006) or 24 h if excessive muscle damage occurred (Akimoto et al. 2002), which is, however, unlikely in swim training. Therefore, it is to be expected that we cannot measure these acute increases of sICAM-1 in response to a training stimulus when analyzing basal chronic changes at rest. In contrast, more permanent changes in populations of circulating lymphocytes are targets of this regulation and more likely to occur as a result of chronic alterations in physical stress. Consequently, these changes stay consistent over longer periods of time, if the physical stress is not acutely altered. Therefore, in contrast to training studies in clinical populations, which observed a decrease in sICAM-1 (Aksoy et al. 2015; Kargarfard et al. 2016), there were no changes observed when analyzing concentrations of sICAM-1 at rest over a training season in athletes. However, changes in lymphocytes as a result of immediate changes in soluble factors like sICAM-1 in response to a training session were detected in this study.

Interestingly, despite no chronic changes having been observed in sICAM-1, changes in proportions of memory cells were negatively correlated with changes in sICAM-1 over the complete training period and in the second nine weeks of training. Moreover, a positive association between changes in proportions of naïve cells and changes of sICAM-1 was observed in the second nine weeks of training. Previous studies have shown ICAM-1 to be important for the transendothelial migration of T cells, however, there is some redundancy in the process, as other molecules like ICAM-2 and VCAM-1 also enable this migration to a certain extent (Boscacci et al. 2010; Lehmann et al. 2015; Reiss and Engelhardt 1999). Furthermore, cell expression of LFA-1 is also an important factor for T-cell migration (Shulman et al. 2009). Our findings possibly indicate that the expression of ICAM-1 and serum concentrations of sICAM-1 may play an important role in the maturation and differentiation of CD4^+^ cells from naïve to memory cells. Furthermore, increased concentrations of sICAM-1 in the blood may reduce the extravasation of naïve CD4^+^ (CD45RA^+^ CD45RO^−^ CD4^+^) cells, by competitively binding to ICAM-1 receptors. The extravasation process is, however, necessary, as the maturation process occurs in peripheral tissue and organs, where naïve CD4^+^ (CD45RA^+^ CD45RO^−^ CD4^+^) cells encounter their specific antigen and can differentiate to effector and memory status.

## Perspective

Current literature shows the distribution and function of different subclasses of lymphocytes to be essentially altered in response to exercise (Walsh et al. 2011). Our study supports these findings and further shows NK cells to be particularly sensitive to prolonged periods of high training volumes. Whether reduced proportions of circulating NK cells as a result of high training volumes also alter NK cell function needs to be further investigated. Furthermore, our study is unique in the fact that it aimed to investigate the underlying mechanisms for the altered composition of circulating T and NK cells over a 16-week training period in athletes. While concentrations of sICAM-1 did not statistically change over the training period, maturation and migration of CD4^+^ cells seems to be associated with altered sICAM-1 concentrations in athletes. Further research is needed to elucidate specific mechanisms for exercise-induced lymphocyte trafficking and also investigate the effects of these changes on immune function.

## Data Availability

The datasets are available from the corresponding author on reasonable request.

## Abbreviations

FACS: Fluorescence activated cell sorting
IL-6: Interleukine 6
LFA-1: Lymphocyte function associated antigen 1
MID: Middle distance swimmers
NK: Natural killer cells
RPE: Rate of perceived exertion
sICAM-1: Soluble intercellular adhesion molecule 1
SS: Sprint swimmers
Treg: Regulatory T cells
VCAM-1: Vascular cell adhesion molecule 1
Vmax: Maximal swimming speed

## References

[CR1] Akimoto T, Furudate M, Saitoh M, Sugiura K, Waku T, Akama T, Kono I (2002). Increased plasma concentrations of intercellular adhesion molecule-1 after strenuous exercise associated with muscle damage. Eur J Appl Physiol.

[CR2] Aksoy S, Findikoglu G, Ardic F, Rota S, Dursunoglu D (2015). Effect of 10-week supervised moderate-intensity intermittent vs. continuous aerobic exercise programs on vascular adhesion molecules in patients with heart failure. Am J Phys Med Rehabil.

[CR3] Bartzeliotou AI, Margeli AP, Tsironi M, Skenderi K, Bacoula C, Chrousos GP, Papassotiriou I (2007). Circulating levels of adhesion molecules and markers of endothelial activation in acute inflammation induced by prolonged brisk exercise. Clin Biochem.

[CR4] Boscacci T, Pfeiffer F, Gollmer K, Isabel A, Sevilla C, Martin AM, Soriano SF, Natale D, Henrickson S, Von Andrian UH, Fukui Y, Mellado M, Deutsch U, Engelhardt B, Stein JV (2010). Comprehensive analysis of lymph node stroma-expressed Ig superfamily members reveals redundant and nonredundant roles for ICAM-1, ICAM-2, and VCAM-1 in lymphocyte homing. Blood.

[CR5] Constantin G, Majeed M, Giagulli C, Piccio L, Kim JY, Butcher EC, Laudanna C (2000). Chemokines trigger immediate β2 integrin affinity and mobility changes: differential regulation and roles in lymphocyte arrest under flow. Immunity.

[CR6] Cosgrove C, Galloway SDR, Neal C, Hunter AM, McFarlin BK, Spielmann G, Simpson RJ (2012). The impact of 6-month training preparation for an Ironman triathlon on the proportions of naive, memory and senescent T cells in resting blood. Eur J Appl Physiol.

[CR7] Dhabhar FS (2014). Effects of stress on immune function: the good, the bad, and the beautiful. Immunol Res.

[CR8] Fischer CP (2006). Interleukin-6 in acute exercise and training: what is the biological relevance ?. Exerc Immunol Rev.

[CR9] Foster C, Hector LL, Welsh R, Schrager M, Green MA, Snyder AC (1995). Effects of specific versus cross-training on running performance. Eur J Appl Physiol.

[CR10] Gleeson M, McDonald WA, Cripps AW, Pyne DB, Clancy RL, Fricker PA (1995). The effect on immunity of long-term intensive training in elite swimmers. Clin Exp Immunol.

[CR11] Gleeson M, McDonald WA, Pyne DB, Clancy RL, Cripps AW, Francis JL, Fricker PA (2000). Immune status and respiratory illness for elite swimmers during a 12-week training cycle. Int J Sports Med.

[CR12] Hubbard AK, Rothlein R (2000). Intercellular adhesion molecule-1 (ICAM-1) expression and cell signalling cascades. Free Radical Biol Med.

[CR13] Kargarfard M, Lam ETC, Shariat A, Asle Mohammadi M, Afrasiabi S, Shaw I, Shaw BS (2016). Effects of endurance and high intensity training on ICAM-1 and VCAM-1 levels and arterial pressure in obese and normal weight adolescents. Phys Sportsmed.

[CR14] Krüger K, Mooren FC (2007). T cell homing and exercise. Exerc Immunol Rev.

[CR15] Krüger K, Lechtermann A, Fobker M, Völker K, Mooren FC (2007). Exercise-induced redistribution of T lymphocytes is regulated by adrenergic mechanisms. Brain Behav Immun.

[CR16] Leeuwenberg JF, Smeets EF, Neefjes JJ, Shaffer MA, Cinek T, Jeunhomme TM, Ahern TJ, Buurman WA (1992). E-selectinand intercellular adhesion molecule-1 are released by activated human endothelial cells in vitro. Immunology.

[CR17] Lehmann JCU, Jablonski-westrich D, Springer T, Springer T, Hamann A (2015). Overlapping and selective roles of endothelial intercellular adhesion molecule-1 (ICAM-1) and ICAM-2 in lymphocyte trafficking. J Immunol.

[CR18] Mujika I, Chatard JC, Geyssant A (1996). Effects of training and taper on blood leucocyte populations in competitive swimmers: relationships with cortisol and performance. Int J Sports Med.

[CR19] Nielsen HG, Lyberg T (2004). Long-distance running modulates the expression of leucocyte and endothelial adhesion molecules. Scand J Immunol.

[CR20] Pedersen B, Hoffman-Goetz L (2000). Exercise and the immune system: Regulation, integration, and adaptation. Physiol Rev.

[CR21] Pedersen L, Idorn M, Pedersen BK, Straten P, Hojman P, Pedersen L, Idorn M, Olofsson GH, Lauenborg B, Nookaew I, Hansen RH (2016). Voluntary running suppresses tumor growth through epinephrine- and IL-6-dependent NK cell mobilization and redistribution. Cell Metab.

[CR22] Prieto-Hinojosa A, Knight A, Compton C, Gleeson M, Travers PJ (2014). Reduced thymic output in elite athletes. Brain Behav Immun.

[CR23] Rama L, Teixeira AM, Matos A, Borges G, Henriques A, Gleeson M, Pedreiro S, Filaire E, Alves F, Paiva A (2013). Changes in natural killer cell subpopulations over a winter training season in elite swimmers. Eur J Appl Physiol.

[CR24] Rehman J, Mills PJ, Carter SM, Chou J, Thomas J, Maisel AS (1997). Dynamic exercise leads to an increase in circulating ICAM-1: further evidence for adrenergic modulation of cell adhesion. Brain Behav Immun.

[CR25] Reiss Y, Engelhardt B (1999). T cell interaction with ICAM-1-deficient endothelium in vitro: transendothelial migration of different T cell populations is mediated by endothelial ICAM-1 and ICAM-2. Int Immunol.

[CR26] Rosenthal R, DiMatteo MR (2001). Meta-analysis: recent developments in quantitative methods for literature reviews. Annu Rev Psychol.

[CR27] Seiler KS, Kjerland GØ (2006). Quantifying training intensity distribution in elite endurance athletes: Is there evidence for an “optimal” distribution?. Scand J Med Sci Sports.

[CR28] Shephard RJ (2003). Adhesion molecules, catecholamines and leucocyte redistribution during and following exercise. Sports Med.

[CR29] Shephard RJ, Shek PN (1999). Effects of exercise and training on natural killer cell counts and cytolytic activity: a meta-analysis. Sports Med.

[CR30] Shulman Z, Shinder V, Klein E, Grabovsky V, Yeger O, Geron E, Montresor A, Bolomini-Vittori M, Feigelson SW, Kirchhausen T, Laudanna C, Shakhar G, Alon R (2009). Lymphocyte crawling and transendothelial migration require chemokine triggering of high-affinity LFA-1 integrin. Immunity.

[CR31] Simpson RJ, Florida-James GD, Whyte GP, Guy K (2006). The effects of intensive, moderate and downhill treadmill running on human blood lymphocytes expressing the adhesion/activation molecules CD54 (ICAM-1), CD18 (β2 integrin) and CD53. Eur J Appl Physiol.

[CR32] Simpson RJ, Florida-James GD, Cosgrove C, Whyte GP, Macrae S, Pircher H, Guy K (2007). High-intensity exercise elicits the mobilization of senescent T lymphocytes into the peripheral blood compartment in human subjects. J Appl Physiol.

[CR33] Steensberg A, Febbraio MA, Osada T, Schjerling P, Van Hall G, Saltin B, Pedersen BK (2001). Interleukin-6 production in contracting human skeletal muscle is influenced by pre-exercise muscle glycogen content. J Physiol.

[CR34] Teixeira AM, Rama L, Carvalho HM, Borges G, Carvalheiro T, Gleeson M, Alves F, Trindade H, Paiva A (2014). Changes in naïve and memory T-cells in elite swimmers during a winter training season. Brain Behav Immun.

[CR35] Walsh NP, Gleeson MM, Shephard RJ, Gleeson MM, Woods JA, Bishop NC, Fleshner M, Green C, Pedersen BK, Hoffman-Goetz L, Rogers CJ, Northoff H, Abbasi A, Simon P, Immunol- E (2011). Institutional Repository Position statement part one: immune function and exercise. Exerc Immunol Rev.

[CR36] Weinhold M, Shimabukuro-Vornhagen A, Franke A, Theurich S, Wahl P, Hallek M, Schmidt A, Schinköthe T, Mester J, Von Bergwelt-Baildon M, Bloch W (2016). Physical exercise modulates the homeostasis of human regulatory T cells. J Allergy Clin Immunol.

[CR37] Wenning P, Kreutz T, Schmidt A, Opitz D, Graf C, Voss S, Bloch W, Brixius K (2013). Endurance exercise alters cellular immune status and resistin concentrations in men suffering from non-insulin-dependent type 2 diabetes. Exp Clin Endocrinol Diabetes.

[CR38] Witard OC, Turner JE, Jackman SR, Tipton KD, Jeukendrup AE, Kies AK, Bosch JA (2012). High-intensity training reduces CD8+ T-cell redistribution in response to exercise. Med Sci Sports Exerc.

